# Sensory professionals’ perspective on the possibilities of using facial expression analysis in sensory and consumer research

**DOI:** 10.1002/fsn3.2393

**Published:** 2021-06-13

**Authors:** Ulriikka Savela‐Huovinen, Auli Toom, Antti Knaapila, Hanni Muukkonen

**Affiliations:** ^1^ Department of Economics and Management Faculty of Agriculture and Forestry University of Helsinki Helsinki Finland; ^2^ Centre for University Teaching and Learning Faculty of Educational Sciences University of Helsinki Helsinki Finland; ^3^ Department of Food and Nutrition Faculty of Agriculture and Forestry University of Helsinki Helsinki Finland; ^4^ Faculty of Education University of Oulu Oulu Finland

**Keywords:** data, emotions, facial expression analysis, skills

## Abstract

The increase in digitalization, software applications, and computing power has widened the variety of tools with which to collect and analyze sensory data. As these changes continue to take place, examining new skills required among sensory professionals is needed. The aim with this study was to answer the following questions: (a) How did sensory professionals perceive the opportunities to utilize facial expression analysis in sensory evaluation work? (b) What skills did the sensory professionals describe they needed when utilizing facial expression analysis? Twenty‐two sensory professionals from various food companies and universities were interviewed by using semistructural thematic interviews to map development intentions from facial expression recognition data as well as to describe the established skills that were needed. Participants’ facial expressions were first elicited by an odor sample during a sensory evaluation task. The evaluation was video recorded to characterize a facial expression software response (FaceReader™). The participants were interviewed regarding their opinions of the data analysis the software produced. The study findings demonstrate how using facial expression analysis contains personal and field‐specific perspectives. Recognizability, associativity, reflectivity, reliability, and suitability were perceived as a personal perspective. From the field‐specific perspective, professionals considered the received data valuable only if they had skills to interpret and utilize it. There is a need for an increase in training not only in IT, mathematics, statistics, and problem‐solving, but also in skills related to self‐management and ethical responsibility.

## Graphical abstract

This research paper will provide an understanding of how to utilize facial expression data analysis. The study results should be considered when planning to take facial expression software to practical sensory evaluation work.

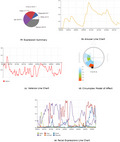

## 1 INTRODUCTION

The food industries are facing new demands on skills and knowledge due to an increase in digitalization. The employers have reported difficulties predicting the required skills and competencies, and graduates do not fully meet their expectations and requirements for a rapidly changing sector (Flynn et al., 2013; Weston et al., 2019). When working with digital technologies and data rather than traditional measures of products, new expectations for sensory professionals are likely to emerge, which challenge the traditional skills in sensory and consumer research. However, there is limited knowledge on food sensory professionals’ perspective of work changes and necessary skills. Since digitalization has widened the variety of sensory methods to be performed by the sensory professionals, continual development to acquire the necessary skills should begin by identifying the skills needed.

### 1.1 Using facial expression measurement of emotion responses in sensory evaluation

Facial measurement of product‐evoked emotions can provide useful and applicable data from consumer emotions (Abbasi et al., 2013; Meiselman, 2016). There are a few software packages to measure facial expression of emotions. Several studies have been published about the measurement of automated facial expression analysis (AFEA) tools (FaceReader™, PrEmo, Affdex), and a few of them using FaceReader™ technology (e.g., De Wijk et al., 2012; Danner et al., 2014; Zhi et al., 2018). The software measures facial expressions related to emotions based on standardized facial movement coding system, the Facial Action Coding System (FACS) developed by Ekman et al. (1978).

A Dutch company named Noldus has developed the FaceReader™ software, which analyzes facial expression patterns, whether from recorded videos or in a real‐time video stream. The FaceReader™'s facial expression analysis is an emotion indicator, and it is noninvasive and acquired through video recording (Zhi et al., 2018). It also reconstructs the face in a three‐dimensional space, based on a 500‐point finite element model. Facial measurement is based on a discrete theory of universally recognized emotions (i.e., anger, disgust, contempt, fear, happiness, sadness, and surprise) (Ekman & Cordaro, 2011). It can be helpful in finding rapid, uncontrollable responses that are related to liking and preferences. Exploring emotion patterns elicited by varying stimuli provides additional value and insights in understanding emotions in relation to verbal expressions of emotion and acceptability (Leitch et al., 2015).

Facial expression measurement technology has been used for research in fields such as psychology, education, and consumer behavior (Rocha et al., 2019). Several studies of advantages and disadvantages, difficulties related to use, and the reliability and validity of the method have been conducted. The robustness and reliability of FaceReader™ have been tested. According to Terzis et al. (2010), the emotions it measured agreed with the judgments of trained observers on up to 89% of occasions. Measuring emotions evoked by a wide variety of eliciting conditions, including statements that referred directly to sensory properties and experienced consequences, and statements that referred to more indirect conditions, such as expectations, recognition, and associations have been reported. However, according to Hwang and Matsumoto (2016), there were difficulties in using FaceReader™ related to training, technical and methodological aspects and to the context of the situation. Their study pointed out that expressed emotional responses can occur from the product but also from the evaluation process, environment, and the evaluators’ behavior and circumstances. Reasons for facial expression can vary and enjoyment of product is not always detectable on the face (Hwang & Matsumoto, 2016). Generally, a product can elicit many subjective emotions and multiple emotions, rather than eliciting a single emotion, and individuals differ in handling their emotional responses (Desmet & Schifferstein, 2008). More positive than negative emotions were experienced when the product is owned, is used precisely for pleasure, and if it fulfilled a consumer's goals (Schifferstein & Desmet, 2010). When measuring food‐related emotions, the variability in emotional response across and within participants and incomplete understanding of the evoked emotion terms affected the consistency of the results (Köster, 2003).

During the FaceReader™'s video recording, staying still is recommended and participant's face should not be covered by anything (Danner et al., 2014). Moreover, awareness of video recording while experiencing the sample could introduce a bias among participants and they might try to limit their expressions. To avoid misinterpreting the results, extra care is needed when using the software and undertaking analysis (Rocha et al., 2019) and interpretations.

Arousal and valence are dimensions describing emotions evoked from human consciousness, and each emotion can be described according to its position on the pleasantness and arousal dimensions (Barrett, 2016; Russell, 1980). According to Russell and Barrett (1999), valence (positive or negative) and arousal (involves activation or deactivation) congregate emotions in two fundamental dimensions. FaceReader™'s circumplex model is based on Russell’s (1980) “circumplex” model of emotions, in which all measured emotions are placed into a two‐dimensional space.

### 1.2 Skills of sensory evaluation and consumer research

Digitalized instruments and methods give rise to new requirements for skills. When the changes in the field continue to take place, the examination of skills needed allows to identify the new skills required by sensory professionals. Skills have a variety of definitions, referring to an acquired ability or capacity. According to Eraut (2000, p.114), skills are part of a particular type of knowledge, allowing representations of competence, capability, or expertise in which the use of such skills and propositional knowledge are closely integrated. Skills also refer to highly competent performance (Smith, 2002). In a general approach, skill means “the ability to apply knowledge and use know‐how to complete tasks and solve problems” (European Commission, 2008, p. 11). Effective and progressive problem‐solving skills are an expert's capacity to strive continuously for a higher level where a problem can be approached. Adaptive expertise is a continuous, active search for opportunities to develop knowledge and understanding (Hatano & Inagaki, 1992). When new technologies and methods are introduced, they will always require employees to learn new skills.

Sensory evaluation requires individual physiological and cognitive skills. According to earlier studies, the skills required are sensory analysis methods and decision‐making, teamwork capabilities, and problem‐solving (Lawless & Klein, 1989; Savela‐Huovinen et al., 2018; Stone et al., 2012). According to Flynn et al. (2013), the most desired food‐sector skill in the field of research and development is product development. Communication skills have been defined as highly desired generic skills, followed by thinking and problem‐solving and skills demonstrating positive attitudes and behavior (Flynn et al., 2013). In sensory evaluation, the interactions between novel methods and procedures with traditional product test variables and the number of new applications emphasize the skills required to understand the methodological issues and problems in product emotion research (Jager and Cardello, 2016).

Levy and Murnane (2004) categorized occupational tasks as *changing by digitalization* where one category includes tasks that require problem‐solving capabilities, intuition, creativity, and persuasion. These tasks are characteristic in professional, technical, and managerial occupations with high levels of education and analytical capability, and place a premium on inductive reasoning, communications ability, and expert mastery (Levy & Murnane, 2004). The ability to communicate, acquire information, and think critically was included in formal education programs in food science (Flynn et al., 2013; LeGrand et al., 2017).

### 1.3 Research questions

The aim with the study was to obtain information from sensory professionals on how they experienced the use of FaceReader™ in their work. We wanted to gain an in‐depth knowledge of this issue from the sensory professional's perspective. The objective was to gain understanding of how the sensory professionals, who are experts of their field, experienced the possibilities of the software in relation to their work and more broadly in relation to the field. And further, how facial expression analysis can be used in sensory and consumer research. The sensory professionals were not experienced with facial expression analysis; therefore, their responses can be understood as potential uses or expectations based on experiences gained through participation in the study. The specific research questions were:

1. How did sensory professionals perceive the option of using facial expression analysis in sensory evaluation?
2. What skills did the sensory professionals describe they would need when utilizing facial expression analysis?

## 2 Materials and method

### 2.1 Overview

The qualitative study was based on a semistructured thematic interview about the experiences of evaluating and conducting facial expression analysis. Sensory evaluation experiments with facial expression analysis software were conducted in a sensory laboratory at the University of Helsinki. These met the standard requirements (ISO8589, 2007) to control and standardize the environmental conditions for sensory evaluation and video recording. The evaluation was video recorded and characterized by the FaceReader™7.1 software (Noldus Information Technology). The semistructured interviews with the data outputs were conducted next to the laboratory.

The University of Helsinki Ethical Review Board in humanities, social and behavioral sciences evaluated the research plan of the study. It stated that the study followed the Ethical principles of research issued by the Finnish Advisory Board on Research Integrity and was ethically acceptable (Statement no. 30/2018).

### 2.2 Participants and recruitment

Twenty‐two participants from Finnish food companies and universities were interviewed to gain an understanding of how facial expressions and analyses can be utilized in sensory and consumer research. The invitation to take part in the research was sent out via email to the members of the Finnish Society of Food Science and Technology in May 2018 (*n* = 1,160). We estimated that about one‐third of the email recipients work in the sensory science field. The study was carried out with the assessors who volunteered and were equivalent to standardized expert sensory assessors (ISO 5492, 2008). The criterion for selecting the participants was that the participant had to work in a sensory laboratory or product development department as an assessor. The participants who agreed with the requirements were involved in the experiments. They signed an informed consent form agreeing that the video data, FaceReader™ analysis data, self‐report, and interview data could be used for research purposes. No previous experiences with the FaceReader™ software were reported by the participants. Participants’ demographic information is shown in Table 1.

**TABLE 1:** Participants’ background information

Number of participants	22
Average age	38.3 years (*SD* = 9.3)
Gender	20 females 2 males
Worked in	
large company (>101)	63.6%
middle‐sized company (21–100)	18.2%
small company (<20)	18.2%
Master or doctoral degree	91.0%
Bachelor's degree	9.0%
≤5 years of work experience	40.9%
6–20 years of work experience	50.0%
>20 years of work experience	9.1%

### 2.3 Samples and self‐reports

In this study, facial expressions were elicited by odor samples. The participants evaluated the samples for pleasantness, intensity, and familiarity on visual analogue scales (10‐cm line scales printed on paper). After scaling, the participants described the quality of the odor with their own words (they were asked to write 1–5 descriptive words/sample). The samples were: sea buckthorn juice (20% in tap water), rapeseed oil with garlic (100%), balsamic vinegar (100%), and l‐carvone, d‐carvone, and beta‐ionone (10%), all diluted with propylene glycol (>99.5%; Sigma Aldrich). Selection of the flavoring ingredients and odorants was based on their ability to produce an adequate odor. Water was provided for the participants to neutralize the sense of smell before sniffing the sample. The order of the samples was randomized but it was kept same for all evaluators, because in this study our focus was on the experiences of the assessors, not on the properties of the samples. Self‐reports were requested after evaluation of each sample.

### 2.4 Procedure

First, the participants were instructed (but not extensively trained) for the evaluation. General knowledge about the technical specifications of the FaceReader™ software was provided to the participants, and also what kind of analyses can be carried out (e.g., facial expression classification, valence and arousal calculation). Previous studies were mentioned in relation to the weaknesses and opportunities of the software. Detailed results were not explained because these could have influenced the evaluation process to the opinions of the facial expression analysis given by the professionals.

Before the experiment, they were informed about the general experimental purpose, the use of line scales, the camera recording, and characterization of a facial expression responses by the FaceReader™. The participants were instructed to sniff the sample at once after opening the cap and to remove the odor vial away from face immediately after sniffing. They received the following instructions before the evaluation task:

1. Adjust your position to make it comfortable, as you are not supposed to move during the experiments.
2. There are samples in front of you. Please sniff them one by one and keep facing the camera while sniffing.
3. Remove the vial from your face and look directly ahead. Do not cover your face during the experiments.

No timer was used, to ensure natural, spontaneous facial expressions and to keep the experiment as natural as possible. The self‐reports were collected to a data spreadsheet for further analysis.

### 2.5 Video recording

A high‐resolution digital video camera (Logitech, Brio stream ultra HD4K, 5× optical zoom) was used for video recording. The camera was placed in front of the participants and adjusted to take a straight frontal face view. The participants were instructed to minimize head movement. The light was white and uniform, and the background was also white to ensure the video was in good light condition. The distance of participant from the camera was approximate 30 cm. The video was set at 5–30 frames/s with frame dimension 1,920 × 1,080, and the recordings were saved as AVI files. The recording was started when evaluation started (the evaluation of the last sample was recorded separately) to visualize the facial expressions elicited by the samples. All the recordings were analyzed with face model “Western,” and “continuous calibration” was chosen for standardization.

### 2.6 Semistructured interviews

**FIGURE 1:**
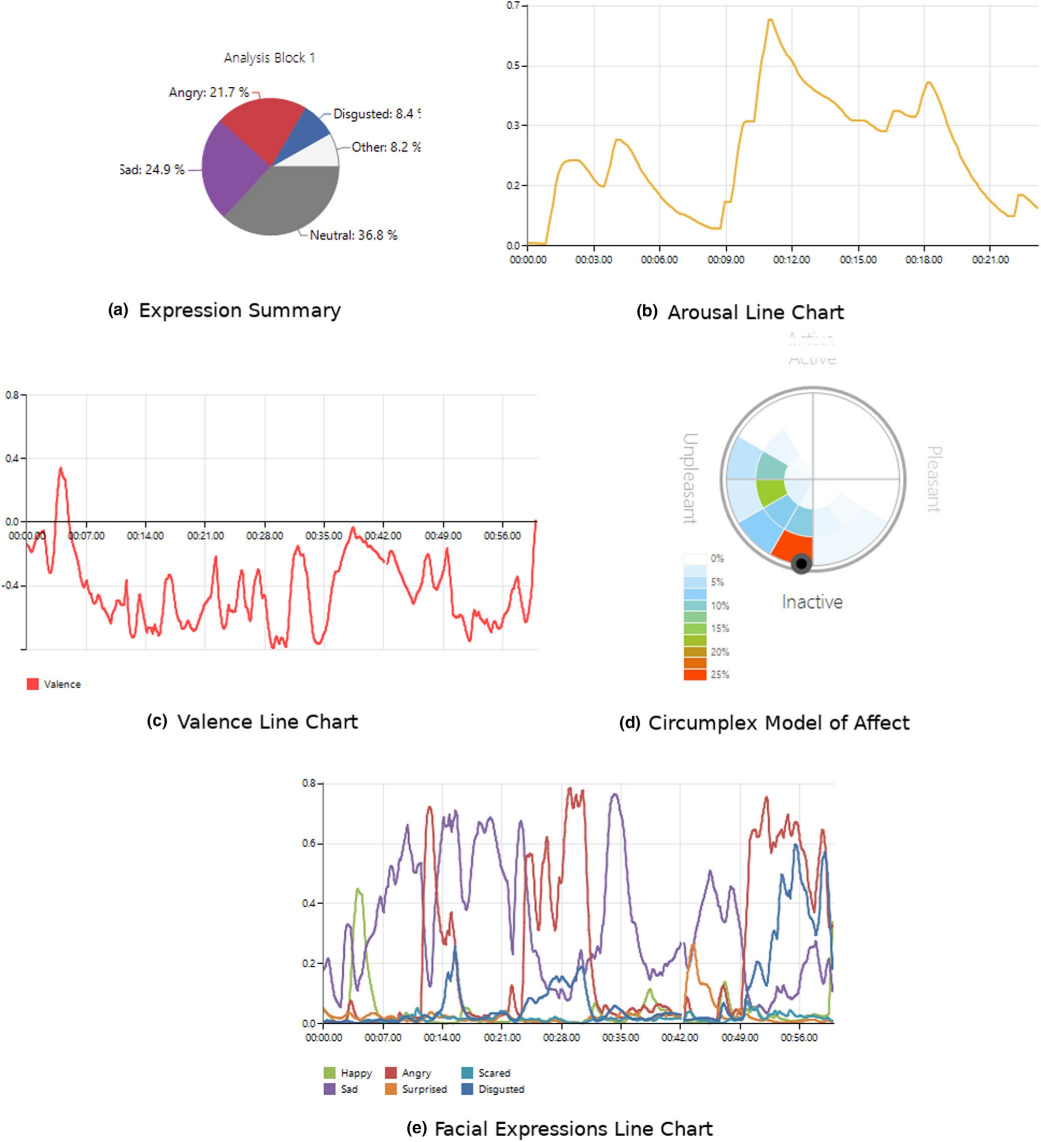
Example graphical figures (a–e) of the FaceReader™ facial expression data analysis

The participants were interviewed individually and in a group of 2–3 people about their experiences of the use of facial expression analysis and the usability of data analysis for one sample (balsamic vinegar). They were asked to describe their experiences as well as the interpretations and utilization of the results. The perceptions were formed in an interview situation in which everyone received their own facial expression printed outputs. The participants were introduced to the outputs, they had opportunity to ask additional questions during the interview, and they explained their perceptions. The participants reviewed the following FaceReader™ outputs: (a) expression summary chart, (b) arousal line chart, (c) valence line chart and (d) circumplex model of affect and (e) facial expression line chart (not provided in the first four interviews). The graphical outputs were color prints, and the participants looked at each photograph individually and then together while expressing their thoughts. The example figures of the data analysis are presented in Figure 1.

The interview themes were: (a) The opportunities to use facial expression analysis in sensory evaluation, (b) The opportunity to use facial expression analysis in creating descriptive vocabulary and (c) Changes related to sensory evaluation work. The interviews were conducted either individually or in groups of two or three. The interviews lasted 30 min each, and were audio recorded and transcribed verbatim. The researcher participated in the situation but did not influence the participants’ answers. The interview themes and questions are presented in detail in Appendix 1.

### 2.7 Data analysis

The data used in the study consisted of the FaceReader™ outputs and transcribed interviews. The self‐reports on odor perception represented a normal evaluation practice and were not analyzed for the study. The interview data were analyzed by following the principles of inductive content analysis, with a focus on how the facial expression analysis could be utilized and which skills did the participants report to need when using facial expression analysis. The data were first analyzed inductively through repeated examination and continuous dialogue. We used ATLAS.ti software in the data analysis (Scientific Software Development GmbH).

The coding process consisted of three phases and 498 quotations were extracted from the data and categorized. In the first phase, we categorized inductively several text segments representing two broad categories of more specifically coded fragments: the participant's perspective and the field‐ specific perspective. The criteria of the two broad category quotes included perspectives on the use of facial expression analysis for the participants themselves (a) as assessors and for (b) the field of sensory professionals in food industries and universities.

The criteria for the subcategories of the assessor's perspective were that the quotes contained aspects regarding the *recognizability of the odor* *(I), associativity of the odor (II) and aspects of reflectivity (III), reliability (IV), and suitability (V)* of the facial expression analysis.

The criteria for subcategories of the field‐specific perspective were that these quotes contained aspects regarding *products* *(I), practices (II), consumer and preference tests (III), consumer behavior (IV), sensory analysis (V), context (VI), data (VII), skills (VIII)*. In the third phase of the data analysis, the reported skills were grouped to the categories. Categories, subcategories, and their criteria are presented in Tables 2, 3, 4.

**TABLE 2:** The participant's perspective of the facial expression analysis

Subcategories	Description	*F*	%	Example quotes from the interviews (translated and transcribed from Finnish)
I Recognizability	Description of the odor identification.	43	19.6	*Well I had happy thoughts. My first delight was that it was a familiar one. That it's always a good thing to recognize and even better if you know how to link it to something. I saw myself at a salad table putting on vinegar or eating vinegar chips. I was in that world right away. Now I am confused about the unpleasantness in the graphics*
II Associativity	Description of associations and memories	21	9.6	*I used to make pickled and canned cucumbers with my boyfriend. I remembered the time he was mixing vinegar, sugar and salt in a saucepan and how the vinegar aroma comes from the saucepan*
III Reflectivity	Description of the experiences where constructing new knowledge and perspectives.	74	33.8	*My experience is that happiness and anger as a result of the analysis are very strong emotions. I guess those are the basic emotions of a human being. These were experienced so strong considering that I only sniffed the balsamic vinegar*
IV Reliability	Description of reliability of the data analysis and the method.	22	10.1	*Perhaps this method is not suitable for all products. For example, if you think about cheeses that are easily associated with negative thoughts based on the aroma, even when cheeses are tasted delicious. Or when you evaluate yogurts, the earthy, sour and milky aromas and flavors need to be taken into account*
V Suitability	Description of suitability of the analysis and results of their own evaluation.	59	26.9	*After all, it is easier to interpret whether the odor was pleasant or not pleasant*.
Total		219	100.0	

**TABLE 3:** The field‐specific perspective for using facial expression analysis

Subcategories	Description	*F*	%	Example of the quotations
I Product	Description of product perspective.	12	4.3	*It might be interesting to test sour candy. How would the analytical graph look like if we compare results between those who like and those who do not. Sour candy often tastes bad but somehow, we still like these*
II Practices	Description of general practical work.	49	17.6	*Odor is easy to test with the camera. You just have to put the sample under your nose. But if you have to taste and chew the sample well then, it's a little difficult. If you try to be in front of the camera and then put something in the mouth. Does the spoon even aim at the mouth?*
III Consumer and preference tests	Description of using the analysis in consumer and preference tests	54	19.3	*When testing food for small children*. *They may not always have words, but not even pressure to make judgments*
IV Consumer behavior	Description of consumer behavior	16	5.8	*If the product does not arouse any kind of emotions for the consumer, then the product is not interesting. This is independent of whether the emotion is positive or negative. If emotions are strong then the consumer has an opinion of the product, and the opinion is important. If a consumer doesn't have any emotions for the product they taste, they hardly ever buy it*
V Analytical sensory analysis	Description of using the analysis in analytical evaluation	20	7.2	*Maintaining a trained sensory panel is time consuming and requires frequent training. This method could speed up that work and make it more specific*
VI Context	Description of contextual perspectives	15	5.4	*When you are member of the panel, there's often a situation in which everyone is evaluating at the same table. Then, there can be social pressure also. You can say: "well, that's fine" under the pressure. But maybe this software could reliably see what a person really thinks afterward*
VII Data	Description of data and value of the data	67	24.0	*I have wondered about these emotional issues. If the result from the study is that your product caused sad feelings more than the competitor's product, how useful are the data? How can companies use the information to their advantage?*
VIII Skills	Description of the required skills	46	16.4	*You need to understand the theory and how that affects to the results. What and how your results should be interpreted?*
Total		279	100.0	

**TABLE 4:** Skills required in using facial expression analysis in sensory and consumer research

Subcategories of skills	Description	*F*	%	Example of the quotations
I Thinking and problem‐solving	Questioning skills, criticality, and interpretation skills	13	30.2	*Well certainly to the interpretation of the results, and what conclusions can be drawn from them*
II IT, mathematics, and statistics	Data handling and statistic. Math skills and reasoning. Knowledge of the methods	12	27.9	*After all, it becomes more technical and one has to increase one's own expertise. One has to know what works for what situation and how to use different technologies*
III Language and communication	Combining and describing skills	3	6.9	*One has to know how to interpret the data and also to open it in writing. Write down what is interpreted from the data*
IV Self‐management	Learning skills	2	4.7	*Let's figure out how this situation differs if you have some another new method. You always need to learn, train, and read to learn new methods*
V Ethicality	Data protection and security‐related skills	2	4.7	*There are the challenges with people's privacy and data protection. If cameras are located on store shelves and the software measures the “look” a consumer has when they choose a product. Then someone is the owner of the data and it is valuable. When it happens to a number of people, will it be done without permission? It's a large amount of paper if you have to ask permission*
VI Sensory evaluation and consumer science	Knowledge of the human nature. Sensory and consumer research and facial expressions of emotions	10	23.3	*You must be a pretty iron‐tight sensory professional for doing interpretations, see the nuances and understand these*
VII Educational training and leadership	Training and visualizing skills	1	2.3	*If we are looking for opportunities for using the data, we need to have abilities to train and guide people with graphs. That is hard! It's easy to say something is not good or bad. But when it comes to guiding and advising, it's not simple and easy*
Total		43	100	

To analyze the inter‐rater agreement of classification, an independent rater classified approximately 10%–15% of the analysis produced. The Kappa coefficient for rater agreement was 0.830 (Cohen's Kappa) for analysis of the participant perspective and the field‐specific perspective, which was considered to represent excellent congruity between the raters (over 0.75 rated as excellent, see Fleiss et al., 1969, p. 281).

## 3 RESULTS

The data analysis included participants’ perceptions of facial expression analysis and the skills required. According to the perceptions, facial expression analysis revealed participant's individual and field‐specific perspectives. The study elaborated on the use of facial expression data analysis from these two perspectives. Subcategories and their descriptions are presented in Tables 2 and 3.

### 3.1 The perspective of participants

The *recognizability of the odor* *(I)* was the first part of the evaluation, and it was significant for the participants. That was also reported several times in the interview (19.6%). Thoughts whether the odor sample could or should be identified in the booth caused uncomfortable feelings for the participants. If the odor was not recognized, it was disturbing, while the odor recognition was considered to be a pleasant surprise. The participants tried to estimate the identification time when the odor expression occurred from the outputs of facial expression analysis. Second, the participants described that they added memories and *associations* *(II)* brought by the odor to the evaluation: the memories from childhood, significant events or the gracious association, and associations from some chemicals. Association has connection for odor recognition and knowledge of the identification process is acquired through association.

Facial expression analysis was *reflected* *(III)* by the participants based on their thoughts and actions in the booth, the self‐reports and facial expression analysis. The reflectivity emerged in one‐third of the responses (33.8%). The participants perceived that the emotions predicted by facial expression analysis sometimes conflicted with their self‐reported judgment. Predictions of emotional states did not always correspond with the self‐reports and contained inexplicable emotions according to the participants. Some of the participants agreed with the results of the facial expression analysis, but some questioned them and found the results differing from their own experience or the self‐report. Additional questions about the reflected emotions were raised and the analyses were compared with each other.

After the reflection part, the participants perception was that the facial expression analysis to be *reliable* *(IV)* when it was identical to the self‐report or what they experienced in the booth. The discrepancy between one's own experience and the facial expression analysis or self‐report caused confusion and distrust. Discussions and understanding the context of odor evaluation increased reliability of the facial expression analysis. According to the participants, the intensity of the odor evaluation affects the facial expression analysis; they argued that it reduces the reliability (e.g., when sniffed only once vs. sniffed several times).

Finally, the participants assessed the *suitability* *(V)* of the facial expression analysis and graphical outputs for their work. The participants’ perception was that the analysis output could be used, and the pie chart was considered the easiest to read. On the other hand, graphic scaling problems were mentioned and challenges of numerical values in practical working life. The participants suggested that the interpretation of the data could not be used without interviewing the individuals. Subcategories and descriptions are presented in Table 2.

### 3.2 Perspective of the field

#### 3.2.1 (I) Product

According to the analysis, the participants reported that the facial expression analysis could be beneficial for evaluating the following *products* *(I)*: vinegar, sour confections, aromas, product from a same product category or with similar properties or evaluation of aftertaste (e.g., fatty products). The participants mentioned that even though they evaluated odor as unpleasant, vinegar was compatible with salad. They also revealed that unpleasant odors are common with various foods (e.g., some cheeses, yogurts, milky or earthy products), and the method is not suitable for evaluating preferences about these kinds of product. Subcategories and descriptions are presented in Table 3.

#### 3.2.2 (II) Practices

The facial expression analysis was perceived as a suitable method for measuring children’ expressions because they are often incapable of describing products using words. The interpretation of *practices* *(II)* could be easier than with traditional methods (self‐reports) but would be still time consuming and laborious according to the participants. The analysis requires systematization, automated solutions, and video viewing while evaluating the product. In practice, the time when the expressions occurred is the most important in a practical way, the participants said. It would require more personalized calibration practices to get a useful result, and reporting practices will change. According to the participants, facial expression analysis would require further development, and the variables of evaluation context should be minimized as much as possible. At present, consumer interviews and tests are practically good enough and there is no need for facial expression analysis. They believed it would not replace any traditional methods yet but might replace it in the future.

At the beginning of the facial expression analysis, the video recorded front‐facing evaluation presented challenges for inexperienced evaluators. Producing an unambiguous summary of overall satisfaction and emotions can also be challenging for companies in the beginning.

#### 3.2.3 (III) Consumer and preference tests

Different ways of using the facial expression data analysis in *consumer and preference tests* *(III)* were defined by the participants. It emerged in 19.1% of responses (e.g., for consumer preference tests on packaging design). The involvement of consumers could be a laborious alternative for companies, and companies specified in consumer research could be concentric users of the software. The opinions varied among the participants, and some considered that the software was not suitable for use in consumer tests, but it would be suitable for home testing if the consumer is well informed and has a camera and an online connection.

#### 3.2.4 (IV) Consumer behavior

According to the study analysis, *consumer behavior* *(IV)* is unique, and emotions and brands influence consumers’ acceptance. A consumer does not necessarily buy any product if it does not arouse any emotions (negative or positive) but the feeling of happiness affects acceptance, participants said. Some consumers want to be surprised, while some of them avoid surprises. Generally, survey responses have a greater and more significant impact on the purchase decision than data of emotions. Geographical location and situations were mentioned as an important aspect when testing individual differences between consumers. Consumers do not always tell everything they know about the product, or they do not have knowledge or sufficient vocabulary (e.g., for descriptive analysis). Children were mentioned as a group in need of special arrangements.

#### 3.2.5 (V) Analytical sensory evaluation

The participants perceived that the facial expression analysis could be used in *analytical sensory evaluation* *(V)*. The method could improve training and maintaining the expertise of trained panels. It was not considered suitable for creating vocabulary or a group test, but it could be useful for quick single testing if an evaluator was also a user of the software. According to the participants, facial expression analysis could offer additional value to the following tests: difference tests, quality control, shelf life tracking, seasonal tasting, taste profile creation, and trained panel evaluation.

#### 3.2.6 (VI) The Context

The participants mentioned that the video recording influences the emotions expressed. Sensory evaluation should be conducted in a genuine *context* *(VI)*. If facial expression analysis is used in the context of group test at work, social pressure will influence evaluation, the participants said.

#### 3.2.7 (VII) The data

The participants pointed out how facial expression analysis *data* *(VII)* analysis could provide additional information, and additional training and knowledge is needed. This emerged in nearly one‐quarter of the responses (24.1%). When analyzing the data, interpretation was mentioned as being significant. The participants considered and mentioned security‐related issues because the analysis contained personal data. Numerical information directly from the software was mentioned as being easy to handle, to store, and to present to the clients, and receiving big data was considered valuable.

The participants brought up the benefits of the facial expression analysis for companies. In particular, they raised questions such as, what feelings are interesting from a commercial point of view? Would the facial expression analysis be useful without a discussion session? What is the correspondence between consumers’ self‐report and occurred facial expressions? Is it relevant or even possible to eliminate anomalies from the analysis? Is it possible to use it for learning about one's own expression of emotion?

#### 3.2.8 (VIII) Skills

According to the study analysis, the following *skills* *(VIII)* were reported in the interview: thinking and problem‐solving (1), IT, mathematics, and statistics (2), language and communication (3) self‐management (4), ethical responsibility (5), principles of sensory evaluation and consumer science (6), educational training and leadership (7). Subcategories and descriptions are presented in Table 3.

### 3.3 Identified skills

During the interview, the participants were asked to describe the skills required when using facial expression analysis. The skills presented by the participants and the defined skills, frequencies, and examples of the quotations are shown in Table 4.

According to the study analysis, the participants mentioned that *thinking and problem‐solving* *(I)* are important for everyday interpretation and conclusion making. This emerged in one‐third of responses (30.2%). *IT, mathematics, and statistics* *(II)* skills are needed to process large volumes of data and for producing relevant results. *Language and communication* *(III)* skills are needed to explain the data to clients and colleagues.

More principled s*elf‐management* *(IV)* is essential for learning new practices and software applications. Being aware of *ethical responsibilities* *(V)* is essential when using a human as an emotion producer and analyzing and storing the data. *Principles of sensory analysis and consumer science* *(VI)* and science of the human nature were reported to be needed for understanding emotions. Finally, *Educational training and leadership* *(VII)* are important in controlling challenges when the new technical tools need to be introduced. The identified skills and the subcategories and descriptions required are presented in Table 4.

## 4 DISCUSSION

The findings demonstrate how using facial expression analysis contains personal and field‐specific perspectives. Recognizability, associativity, reflectivity, reliability, and suitability were perceived as a personal perspective. From the field‐specific perspective, professionals considered received data analysis valuable only if they had skills to interpret and utilize it. The study highlights the potential skills suggested and provided by sensory professionals. We conclude that there is a need to increase training not only in IT, mathematics, statistics, and problem‐solving, but also skills related to self‐management and ethical responsibility. The needed skills were raised by the sensory professionals based on their knowledge and experience of sensory evaluation work.

Odors can evoke associations and memories and enable samples to be identified. The study revealed the combination *of associability* and *recognizability* when using facial expression analysis. Evaluated recognizability aspects such us the time of recognition were valuable to the assessors. The time estimation would not be possible without facial expression technology.

The study revealed how personal perspectives of *reflection* and *reliability* are essential aspects when using the software. The important questions are: should we accept facial expression analysis, and should we see it as *suitable* for use? If the analysis is inconsistent with one's own experience, how can product evaluation be interpreted? This can be seen as consumer's choices that are mostly driven by unconscious mechanisms rather than conscious or rational ones (Dijksterhuis & Smith, 2005; Köster, 2009). Facial expression analysis or other implicit measurements can provide further insights when compared to self‐report. According to Andrejevic and Selwyn (2020), the case is the same with any ‘new’ technology and it is important to reflect and problematize facial recognition technology and to consider any undesirable consequences that might result. Regarding *consumer behavior, context, and product* evaluation, the results from the present study agree with the literature (e.g., Hwang & Matsumoto, 2016) about the difficulties of using facial expression analyses and we agree with the arguments for time consumption and need for training. According to the study, there are undesirable consequences that might result related to ethical considerations.

The logic of measuring emotions to influence decision‐making has a foothold in consumer marketing and the development of personalized advertising (Saltman, 2016). When using facial expression analysis, thinking and problem‐solving skills and IT, mathematics, and statistics were highlighted. Learning, training skills, and ethical responsibility have rarely been mentioned in the literature. Facial recognition technologies can be used to compare data with previously analyzed faces that are already stored on a database that contains large numbers of photographed faces with associated names and other personally identifiable information. When scanning and analyzing facial expressions in order to infer people's moods, emotions, and affective states, this study has significance in pointing out that ethical responsibility should be emphasized as a skill needed in sensory evaluation and consumer research.

### 4.1 Methodological considerations

This qualitative study was done in a single country and with a nonrepresentative set of industries and participants. The results would have been more comprehensive if the study had also used experienced FaceReader™ users instead of beginners only. However, the study focus was on authentic professional reflection to evaluate the facial expression analysis results. We argue that the data collected were valid for studying the phenomenon and answering the study questions, as was the method of using inductive content analysis. As a qualitative study, the most significant results were the development of categorization and the distribution of skills. The conceptualization of the results can be generalized and exploited in other studies.

The approach was developed to measure the perception of odor sensory attributes and the participants were informed that the evaluation was being video recorded. From an ethical perspective, this was mandatory, but it could have introduced a bias with participants reacting according to the principle of social desirability. They may also have limited their expressions. It was also impossible to ensure that the participants were not affected by the laboratory environment or other people next to the booth. People are not always aware of their emotional responses or the conditions that underlie their emotions (Barrett et al., 2007). The eliciting conditions reported have been influenced by the participants’ beliefs or expectations between the odor sample and emotions. The objective approach of this study could be seen as treating the observed participants as a type of a physiological bundle focusing on input and measuring outputs. This aligns with the utilitarian approach not just in the objectiveness of the approach, but also viewing participants as objects of a study to produce knowledge.

In an authentic context, food product would always be tasted, not only sniffed, in order to obtain an overall rating of the product (e.g., preference). In turn, product‐evoked emotions are more difficult to evaluate than sensory attributes (Thomson et al., 2010). In addition, the emotion ratings may have been influenced by the participants’ initial emotional state or by the preceding task (in this case previous odor sample). Sensitizing respondents by having them imagine emotions may have stimulated them to report higher levels of emotional relevance in the actual test. The representativeness of the products tested can be questioned; other types of odor may yield different results. We agree with the literature that more research of testing procedures and the context of evaluation is needed (Danner et al., 2014; Hwang & Matsumoto, 2016).

## 5 CONCLUSION

The study contributes to the explanation of how the sensory professionals described and examined personal and field‐specific perspectives of using facial expression analysis. Future research could examine evaluation context and the consequences for their use in product development. According to various affordances provided by changes experienced, the study results could be utilized by training and education providers in order to develop the profession. When developing their expertise and acquiring new skills and competencies, the professionals would be better prepared to adapt new methods and technologies and effectively manage their workplace challenges.

## INFORMED CONSENT

Written informed consent was obtained from all study participants.

## ETHICAL REVIEW

This study was conducted according to the guidelines laid down in the Declaration of Helsinki, and this study was approved by the Institutional Review Board of Helsinki University (statement number 30/2018).

## CONFLICTS OF INTEREST

The authors declare that there is no conflict of interest that could be perceived as prejudicing the impartiality of the study reported.

## AUTHOR CONTRIBUTION

**Ulriikka Savela‐Huovinen:** Conceptualization (lead); Data curation (lead); Formal analysis (lead); Funding acquisition (lead); Investigation (lead); Methodology (lead); Project administration (lead); Resources (lead); Software (lead); Validation (lead); Visualization (lead); Writing‐original draft (lead); Writing‐review & editing (lead). **Auli Toom:** Conceptualization (equal); Data curation (supporting); Formal analysis (equal); Investigation (supporting); Methodology (lead); Supervision (lead); Validation (supporting); Writing‐original draft (equal); Writing‐review & editing (equal). **Antti Knaapila:** Conceptualization (equal); Investigation (supporting); Resources (supporting); Validation (equal); Writing‐original draft (supporting); Writing‐review & editing (supporting). **Hanni Muukkonen:** Conceptualization (equal); Formal analysis (equal); Investigation (equal); Methodology (supporting); Supervision (lead); Validation (equal); Writing‐original draft (equal); Writing‐review & editing (equal).

## Data Availability

The data that support the findings of this study are available on reasonable request from the corresponding author. The data are not publicly available due to privacy or ethical restrictions.

## APPENDIX 1 The theme interview: Facial expression analysis in sensory evaluation

The themes and the questions

I The opportunities for using facial expression analysis in sensory evaluation

1. Describe the facial expression graphics in front of you. What thoughts do the grafts evoke?
2. Describe how the data could be used in your work?
3. Describe how the data could be used generally in the food industry?
4. In what ways do you think the graphical representation such as arousal, valence, emotional summary could be useful/or not useful?
5. Describe the difficulties in using the facial expression data analysis and graphics?
  II The opportunities for using facial expression analysis in creating a descriptive vocabulary

1. Next, look at your descriptive words about balsamic vinegar in the self‐report. Is it possible to use the data/grafts to create more words for describing the vinegar?
2. What do you think about the evaluation of the sample – pleasantness, strength, or familiarity?
3. Is it possible to create a descriptive vocabulary based on the tests results obtained? (e.g., refine the descriptive words)
4. When comparing this facial expression analysis with a traditional descriptive vocabulary, what would you expect to change if the data analysis was used to create the vocabulary for
  - Consumers
  - Professionals

III Changes related to sensory evaluation work

1. What changes related to sensory evaluation and consumer research will be needed if using facial expression analysis?
2. What skills do professionals need when utilizing facial expression data analysis?
